# Fatty acid methyl ester analysis of *Aspergillus fumigatus* isolated from fruit pulps for biodiesel production using GC-MS spectrometry

**DOI:** 10.1080/21655979.2020.1739379

**Published:** 2020-03-16

**Authors:** Ferruh Asci, Busra Aydin, Gulderen Uysal Akkus, Arzu Unal, Sevim Feyza Erdogmus, Safiye Elif Korcan, Israt Jahan

**Affiliations:** a Department of Molecular Biology and Genetics, Afyon Kocatepe University, Afyonkarahisar, Turkey; b Department of Molecular Biology and Genetics, Usak University, Usak, Turkey; c Department of Chemistry, Afyon Kocatepe University, Afyonkarahisar, Turkey; d Department of Agricultural Biotechnology, Faculty of Agriculture, Igdir University, Igdir, Turkey; e Department of Medical Service and Techniques, Suhut Vocational School of Health Services, Afyonkarahisar Health Science University, Afyonkarahisar, Turkey; f Vocational School of Health Services, Usak University, Usak, Turkey; g Department of Bioengineering, Faculty of Chemical and Metallurgical Engineering, Yildiz Technical University, Istanbul, Turkey

**Keywords:** *Aspergillus fumigates*, fatty Acid Methyl Ester, biodiesel, fungal lipids, GC-MS analysis

## Abstract

In this study, a total of six fungal samples were isolated from apple, strawberry and orange pulp. DNA sequence analysis was used as molecular identification method. ITS region was aligned in DNA sequence analysis, and an algorithm sequence similarity was done using BLAST (Basic Local Alignment Search Tool) program to identify these isolates. All the six isolates were identified as *Aspergillus fumigates*. The total lipid content was varied in the isolates which were ranged from 29.4 to 21.0 mg/100 ml. Moreover, the obtained lipid form mycelium biomass of the isolates was transesterified by a base catalyst. The methyl esters were analyzed by using GC-MS. GC-MS Spectrometry revealed the presence of different fatty acids with long chain (C11:0, C15:0, C17:1, C18:2, C16:1). High efficiency biodiesel can be obtained using long-chain fatty acids. Fatty acid profiles of *A. fumigatus* isolated from different fruit pulps have confirmed its potentiality as well as showed the beneficial utilization of these fatty acids for biodiesel production.

## Graphical Abstract

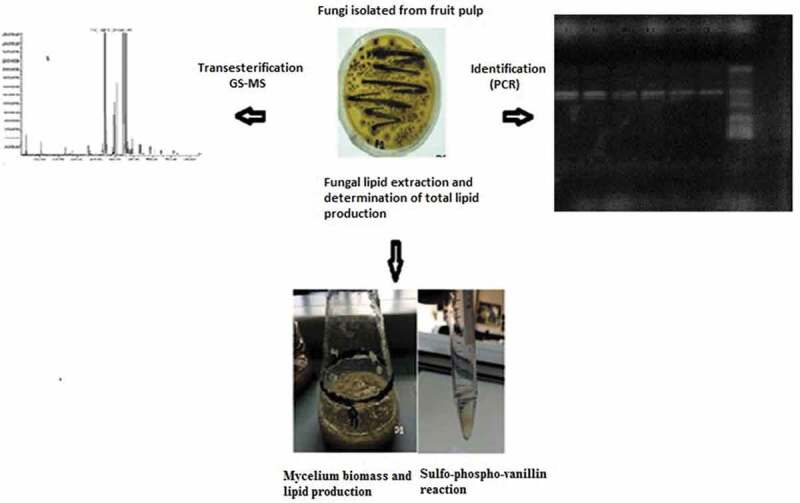

## 1. Introduction

The increasing demand of energy across the world is the result of growing population, economic development, rapid urbanization, process of industrialization, increase of energy-consuming activities, etc. Besides, the advancement of technology offers upgraded products which also require energy [1]. This rising demand compels more energy to be generated from different sources, although fossil fuels contribute to the major energy production and global primary energy consumption. Therefore, finding alternative ways for distributing more energy from the renewable sources can be sustainable, clean and easily available. Biofuels are cost-effective and environment-friendly, renewable energy resources. They are mainly produced by converting the biomass, especially organic substances. Two main types of biofuels are ethanol and biodiesel which have become the most common alternative fuels for transportation, and thereby, replaced traditional nonrenewable fuels like petroleum, diesel and jet fuel [1]. Biodiesel is a promising liquid fuel mainly consists of fatty acid methyl esters (FAMEs), derived from vegetable oils, animal fats, and microbial sources like algae, bacteria and fungi, or recycled cooking grease [1,2]. For the commercial production of biodiesel, it must meet the standards by maintaining essential fuel qualities, i.e. kinematic viscosity, oxidative stability, cetane number (CN), etc. However, these vital properties are associated with the fatty acid methyl esters (FAMEs) profile of biodiesel [3].

The use of microbial systems for the production of nontoxic and biodegradable biodiesel may play a significant role. Researcher has also confirmed that numerous types of fungi are highly efficient to produce biofuels [4]. This is due to the fact that the lipid content in a microorganism exceeds 20% and some fungi accumulate and store high amounts of triacylglycerols (TAGs) [3–7]. Besides, different kinds of fatty acids, such as stearic acid (C18: 0), Oleic acid (C18: 1), linoleic acid (C18: 2), linolenic acid (C18: 3) palmitic acid (C16: 0), and palmitoleic acid (C16: 1) are available in most of the fungi species which can ensure suitable FAMEs profile for standard biodiesel [7,8].

Therefore, filamentous fungi are considered as a suitable and productive feedstock for a sustainable biodiesel production [2,8]. In that case, different filamentous fungi from agricultural wastes, i.e.,, fruit pulp could be the suitable source of fungal fatty acid methyl esters for biodiesel production [2,8,9]. Fruit pulps (FP) are rich in terms of their chemical composition. It has been reported that FPs contain approximately 75% sugar and hemicellulose, 9% cellulose and 5% lignin [10]. Besides, they are easily biodegradable and show a high anaerobic digestion tendency due to having high humidity (75%) [10]. However, unless they are utilized for animal nutrient supplement, a significant portion of fruit pulps are thrown as agricultural wastes. Therefore, they can be utilized as the source of microbes of renewable energy production. Aiming this, it has been targeted in recent years to use the wastes from food industry as a substrate in bioprocess of biofuel production [11]. Nevertheless, very few literatures have focused on the utilization of organic wastes as the source of fungal species and the use of this fungus for microbial biodiesel production since it is quite a new aspect of contemporary science [10,12–15].

The focuses of the present work were to isolate and identify fungal species from fruit pulps, determine the lipid content of these isolates, and finally, investigate the fatty acid methyl ester (FAME) composition after transesterification of fungal oil. According to the literature, this is so far the first study showing that *A. fumigatus* can be isolated from fruit pulps, and at the same time, fatty acids from the isolated fungus are significant for biodiesel production.

## 2. Materials and Methods

### 2.1. Isolation of fungal isolates

Fruit juices were placed in sterile containers, and then the fungal growth was observed at 27 ± 2°C for 14 days. Fruit pulp samples (apple, strawberry and orange pulp) with fungal mycelia were also taken and diluted using the tube dilution method for fungal isolation. In an aseptic condition, 1 gm of fruit sample was transferred into 10 ml of sterile distilled water for dilutions. The dilutions of 10^−4^ and 10^−5^ were spread on malt agar (MA) and potato dextrose agar (PDA) media, and then incubated at 27 ± 2°C for 36 h for multiplying fungal colonies. After the incubation, based on the morphological characteristics, a single colony was isolated from the fully grown fungal colonies cultivated on potato dextrose agar medium. The isolation of fungal colony was performed considering some specific morphologic characteristics, like color, texture appearance, and diameter of the colonies as well as microscopic characteristics as described previously [16].

### 2.2. Extraction of genomic DNA and PCR amplification

All fungal isolates were grown in potato dextrose agar (PDA) medium. A single colony was picked for extraction. DNA extractions of isolates were carried out using the High Pure PCR Template Preparation (Roche) Kit according to the manufacturer’s instructions. PCR amplifications for ITS gene regions were performed. The primers for the amplification were as follows: ITS1 (Forward-IDT) (5’-TCCGTAGGTGAACCTGCGG-3’) and ITS4 (Reverse-IDT) (5’-TCCTCCGCTTATTGATATGC-3’) [17].

PCR amplification process was started by using high fidelity PCR System and dNTPs Pack kit (Roche). The PCR conditions for the amplification of ITS encoding genes consisted of an initial cycle of 2 min at 95°C 1 cycles: denaturation at 94°C for 30 sec 35 cycles, annealing at 55°C for 2 min 35 cycles, and extension at 72°C for 50 sec 35 cycles, and 72°C for 7 min 1 cycle. The amplified products were analyzed on 1.5% agarose gels after completing the PCR cycles. Ethidium bromide (EtBr) was used with agarose gels as the fluorescent tag, and after the electrophoresis, the bands of amplified PCR products were photographed under UV illumination. The isolation and purification of PCR samples from the gel was done by using high pure PCR product purification kit (Roche); and afterward, the sequence analyses were performed by ABI 3100. By using the Basic Local Alignment Search Tool (BLASTN) program, the sequences were compared with the data obtained from the National Center for Biotechnology Information (NCBI) for an algorithm sequence similarity. At the same time, Chromas program was also used for sequence similarity searching.

### 2.3. Mycelium biomass and lipid production

100 ml of medium (Glucose 50 g/L, Yeast extract 1 g/L, KH_2_PO_4_ 5 g/L, NaNO_3_ 2 g/L, MgSO_4_.7H_2_O 0.5 g/L, FeSO_4_.7H_2_O 0.01 g/L) was used for the production of fungal biomass. The stock fungal cultures isolated from fruit pulps were added to the medium under sterile conditions from a suspension of mycelium in 5 mL sterile distilled water. Afterward, the suspension containing medium was placed into an incubator shaker at 27°C, 150 rpm shaking, for 10 days. At the end of the incubation period, the level of lipid production in each fungal culture was investigated.

### 2.4. Fungal lipid extraction and determination of total lipid production

Fungal biomass was collected and kept into the oven at 60°C for allowing it to dry properly. After the drying for sufficient time, the dried biomass was crushed into powder by ceramic mortar, and the dry weight (g) was measured carefully. The resulting dried fungal biomass acquired from different time intervals was used for lipid extraction. Lipid productions were evaluated by a colorimetric method based on the sulfo-phospho-vanillin reaction [18,19]. Vicente et al. [7] investigated the effect of three different solvent systems (chloroform: methanol, chloroform: methanol: water and n-hexane) on lipid extraction from fungus for biodiesel production from *Mucor circinelloides*. Chloroform: methanol was found to be the most effective solvent for extraction [7]. Therefore, Fungal biomass was extracted with 2:1 (v: v) chloroform: methanol mixture following the outcomes of this literature. After the centrifuge, the supernatants were used in lipid production analyses. 0.01 ml of supernatant was dissolved into 1 ml H_2_SO_4_, and incubated into hot water bath for 10 min. At the end of the incubation period, tubes were transferred into a cooling system, and later, 1 ml phospho vanillin indicator and 1 ml distilled water were added with the cooled suspensions. Olive oil was dissolved into the ethanol which was used as a standard solution. Both the standard and the samples were analyzed via spectrophotometer by measuring the rate of increase in absorbance at 530 nm. All experiments were performed with three replications.

### 2.5. Transesterification of Fungal lipids

Transesterification of fungal lipids was performed using the ISO-5509, 2000 method. 15–32 mg of lipid samples and 1 ml n-hexane were added into the eppendorf tube and vortexed properly. Later, 100 µl 2 N methanol-KOH was added, and centrifuged at 300 rpm for 2 min. After the centrifuge, 0.08 mg Na_2_SO_4_ was added into eppendorf tube, and again, centrifuged at 10,000 rpm for 5 min. Afterward, the organic supernatant was transferred into the vials.

### 2.6. Analysis of fatty acid methyl ester on GC-MS spectrometry

Methyl esters which were obtained from the microbial lipid transesterification were identified using GC-MS Spectrometry (Agilent GC-MS 7683B series). Flame ionization detector (FID) and TR-CN100 Teknokroma Capillary Column were used for the analysis. Fatty acid was evaluated according to the methyl ester standard (Comp. FAME Mix 10 mg/ml in CH_2_Cl_2_; Supelco, USA).

## 3. Results and Discussion

In the present study, totally, six fungal samples were isolated from apple pulp (3), strawberry pulp (1), and orange pulp (2). When the internal transcribed spacer (ITS) regions of the isolated fungi were amplified, bands of about 650 bp were obtained. The PCR products were sequenced and analyzed using the BLAST (Basic Local Alignment Search Tool) program. All fungal isolates were identified as *Aspergillus fumigatus* (Table 1). *Aspergillus* species are important in terms of their ability to grow in wide temperature and pH ranges as well as for their tolerance to salts. Therefore, different species of the genus *Aspergillus* are widely used industrially in various vegetable fermentation processes [20].

**Table 1.** Identification of fungal isolates from fruit pulps and total lipid contained of these isolates.

Isolates	Closest gene bank match	Optical density (OD) (SD)	Amount of fat (mg/100 ml)
O_1_	99% *Aspergillus fumigatus*	0.402 (0.0614)	22.7
O_2_	99% *Aspergillus fumigatus*	0.383(0.0510)	21.0
S	100% *Aspergillus fumigatus*	0.475 (0.008)	29.4
A_1_	99% *Aspergillus fumigatus*	0.435(0.0601)	25.7
A_2_	99% *Aspergillus fumigatus*	0.469 (0.0289)	28.8
A_3_	100% *Aspergillus fumigatus*	0.406 (0.0160)	23.1

For fungal lipid production, the fungal biomass was produced using glucose-containing medium. However, the glucose media was adjusted in different pH (4, 5, 7 and 9) and temperature (25°C to 40°C) for observing the optimum growth of fungal mycelium. The best response was observed at 40°C with a pH of 7. Li et al. [21] investigated the microbial lipid production in *Rhodosporidium toruloides* using glucose as the carbon source. The yeast cells showed good growth at 37°C in the presence of 150 g/L glucose and 12 g/L (NH_4_) 2SO_4_ when the medium pH was adjusted to 6 [21].

**Figure 1.**
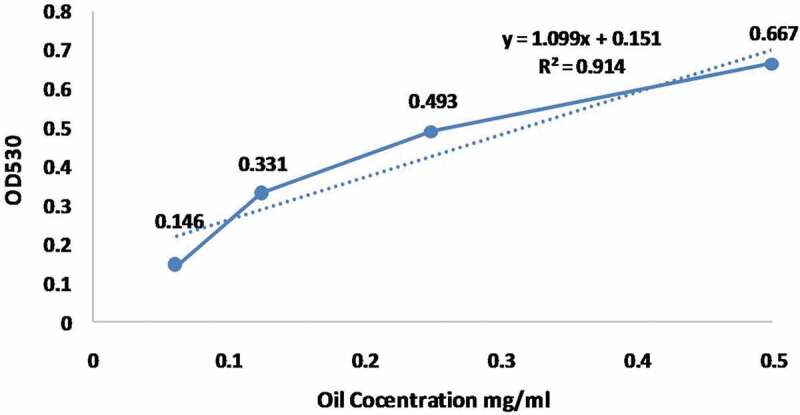
Standard curve of olive oil.

Furthermore, lipid production of isolates was calculated from the olive oil calibration standard curve (Figure 1). Total lipid contains of the fungal isolates are also shown in Table 1. The highest lipid value was determined for the fungal sample of strawberry pulp (S) with the amount of 29.4 mg/100 ml, whereas the lowest value was found in the sample O_2_ (21.0 mg/100 ml) which was isolated from orange pulp. However, the relative rates of all the lipid ingredients among these samples may change due to the development of different fungal stage, age, and culture conditions [22].

Peng and Chen [8] evaluated the lipid content of several fungal species, such as *Microsphaeropsis* sp., *Phomopsis* sp., *Cephalosporium* sp., *Sclerocystis* sp. *Nigrospora* sp. and *Mortierella ramanniana*. The lipid contents of were found as 7.51%, 5.12%, 6.98%, 5.90%, 5.87%, 6.95%, respectively, [8]. Another study by Sergeeva et al. [23] also confirmed the production of a significant amount of lipid (dominated by oleic acid constituted 50% of total fatty acids) from the fungus *Cunninghamella japonica* [23].

**Figure 2.**
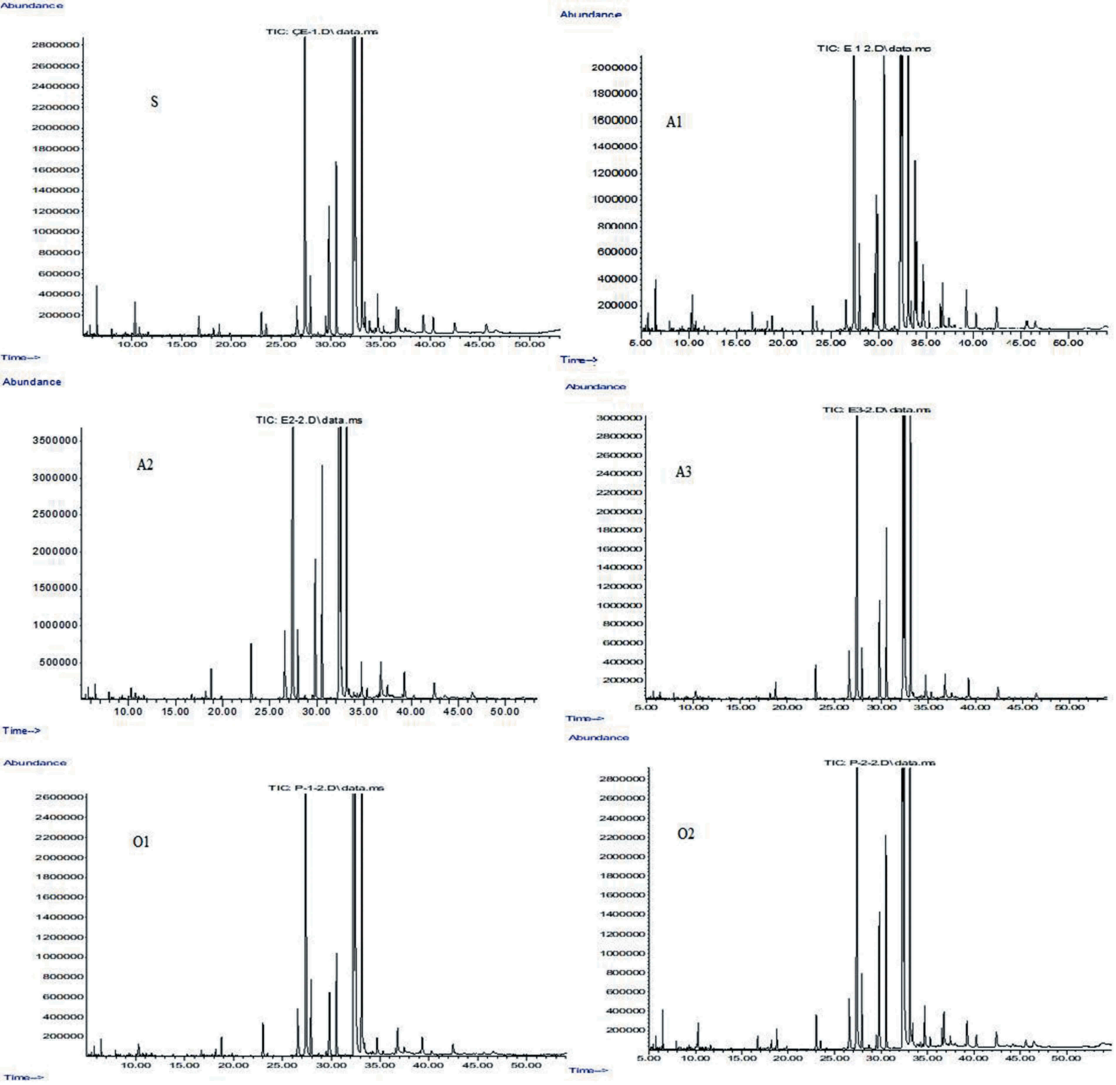
GC-MS chromatograms of methyl esters obtained by transesterification of isolates.

On the other hand, in this study, methyl esters were also obtained from the microbial lipid transesterification of fungal lipids, and the transesterified fatty acids were identified using GC-MS Spectrometry (Figure 2 and Table 2). The fatty acid profile of all isolates confirmed the presence of long-chain fatty acids. The O_1_ isolate contains a high proportion of long-chain fatty acids, 8,11-octadecadienoic acid and 4,7-octadecadienoic acids (C18: 2). The ratios of 8,11-octadecadienoic acid and 4,7-octadecadienoic acid contained in this isolate are 32.245% and 12.680%, respectively. In addition, this isolate contains a high amount of pentadecanoic acid (C15: 0) (17.368%), which is one of the saturated fatty acids. O_2_ isolate also have a high level of 8, 11-octadecadienoic acid (C18:2) (30.675%). Besides, hexadecenoic acid (C16:1) (16.232%), and 5, 9-octadecadienoic acid (C18:2) (13.294%) were also found in this isolates. On the other hand, the extracts of A_1_ contain the highest amount of 9,12-octadecadienoic acid (25.347%), along with 4,7-octadecadienoic acid (14.305%) and hexadecenoic acid (C16:1) (13.165%). Similarly, A_3_ isolates also showed the highest amount of 9, 12-octadecadienoic acid (47.258%), coupled with pentadecanoic acid (19.385%) and 6, 8-octadecadienoic acid (17.266%). On the other side, the fungal sample A_2_ possessed the highest amount of 8, 11-octadecadienoic acid with a percentage of 36.204. Significant amount of Hexadecenoic acid (16.961%) and 6, 8-Octadecadienoic acid (13.435%) were also found using GC-MS analysis of A_2_ isolates. Furthermore, S isolate has 8,11-octadecadienoic acid, hexadecenoic acid and 4,7-octadecadienoic acid with a percentage of 27.906%, 12.759% and 11.939%, respectively (Figure 2, Table 2).

**Table 2.** Fatty acid composition and percentage of the extracted total lipids of fungal isolates using GC-MS analysis.

Fatty acid	Number of carbon atoms: Number of double bonds	Quantity (%)					
		O1	O2	A1	A2	A3	S
8,11-octadecadienoic acid	C18:2	32.745	30.675	-	36.204	-	27.906
Pentadecanoic acid	C15:0	17.368	-	-	-	19.385	-
4,7-octadecadienoic acid	C18:2	12.680	16.232	14.305	-	-	11.939
Hexadecenoic acid	C16:1	1.005	-	13.165	16.961	-	12.759
Heptadecanoic acid	C17:1	1.464	2.416	2.589	2.269	2.388	2.400
Cyclopropaneoctanoic acid	C11:0	1.424	2.563	2.175	-	2.382	2.136
5,9-Octadecadienoic acid	C18:2	9.228	13.294	7.180	-	7.961	-
9,12-octadecadienoic acid	C18:2	-	7.810	25.347	-	47.258	-
6,8-Octadienoic acid	C18:2	-	-	-	13.435	17.266	-

A similar study evaluated fungal fatty acid quantity which was found to be palmitic acid (C16:0) 7–23%; palmitoleic acid (C16:1) 1–6%; stearic acid (18:0) 2–6%; oleic acid (C18:1) 8–40%; linolenic acid (C18:3) 4–42% [2]. Moreover, a study related to the production of biodiesel by Subhash and Mohan [6] confirmed the formation of high lipid (22.1%, 2 g dry biomass, 48 h) in the corncob waste liquors (CWL) used as a substrate by using the genus *Aspergillus awamori* [6]. The fatty acid profile of the *Aspergillus awamori* varied according to the substrate. When corn wastes were used as a substrate, myristoleic acid methyl ester (C14: 1n9c), linolenic acid methyl ester (C18: 3n3) and erucic acid methyl ester (C22: 1n9) were found as fatty acids. When Sabourauds dextrose was used as a broth medium substrate, it was found that the fatty acids were myristoleic acid methyl ester (C14: 1n9c), Cis-11-eicosenoic acid methyl ester (C20: 1), and erucic acid methyl ester (C22: 1n9) [6]. Abu-Elreesh and Abd-El-Haleem [4] conducted an experiment on several fungal isolates collected from different Egyptians ecosystems and assessed their capability of accumulating intracellular lipids [5]. Four fungal isolates with the lipid content from 46% to 71% were identified as promising lipid producers. Gas chromatography–mass spectrometry analysis of produced fatty acids of all isolates revealed the presence of long-chain fatty acids with hexadecanoic acid, octadecanoic acid and 11-octadecenoic acid [5].

Considering these above-mentioned literatures, it can be concluded that *A. fumigatus* isolated from different fruit pulps in this study, is a promising lipid producer. Besides, the fatty acid methyl esters (FAMEs) profile of isolated *A. fumigatus* has also established its potentiality as well as showed the beneficial utilization of these fatty acids for biodiesel production. This is due to the fact that fungi accumulate lipid in the cell during metabolic stress periods, and the main component of the fungal lipid is the triacylglycerides which contain long-chain fatty acids with C_16_ and C_18_ series [24–26]. The presence of long-chain fatty acids of FAME profile improves biodiesel properties, and hence, confirms high fuel efficiency [7,19,27,28].

Use of different filamentous fungus like *Aspergillus* spp. for the production of inexpensive and biodegradable biodiesel has also gained huge attention in recent years. Researchers have demonstrated that a large amount of standard biodiesel can be obtained from oleaginous microorganisms which could be utilized commercially [29,30]. Moreover, it has also investigated that *Candida, Rhodotorula, Aspergillus, Paecilomyces, Fusarium, hormoconis, Penicillium* and *Alternaria* are the potential in biodiesel production [28–32].

From the economic point of view, the potentiality of an organism as a lipid producer is interrelated to the capability of substrate utilization. Fungi can be cultivated easily in carbon-rich medium where they accumulate lipids by converting the carbon-containing compounds [5]. In this context, numerous fungi should be screened particularly for biodiesel production by utilizing cheaper wastes [6,28]. Therefore, the present study was accomplished by isolating *Aspergillus fumigatus* form fruit pulps, and investigating total lipid content and fatty acid methyl ester (FAME) composition of these isolates. Perhaps, this could be the first attempt at screening filamentous fungus from fruit pulps in order to understand their suitability for biodiesel production.

Fuel obtained from a fungus strain through an inexpensive process can be established as an alternative platform that might reduce the future energy crisis. For biodiesel production, the development of microorganisms or improved strains with high lipid content will turn into a potential and promising pathway in the future. The manipulation and regulation of microbial lipid will unlock and widen the opportunities for academic research to demonstrate its potentiality for industrial application in biodiesel production [33–35]. Hence, subsequent studies should be carried out based on the determination of fatty acid profiling using this wastewater as the source of suitable microbial strains. In further, genes of *Aspergillus fumigatus* can be cloned with other *Aspergillus* strains which can be used as industrial hosts for many advanced applications.

## Highlights

- Six fungal samples were isolated from apple, strawberry and orange pulp.
- DNA sequencing identified all the isolates as *Aspergillus fumigates*.
- The fungal lipids form mycelium biomass were transesterified and identified using GC-MS Spectrometry.
- The fatty acid profile of all isolates confirmed the presence of long-chain fatty acids (C11:0, C15:0, C17:1, C18:2, C16:1).
- This is so far the first attempt to utilize fruit pulps for isolating *Aspergillus fumigatus* aiming to evaluate its suitability for biodiesel production.
